# Ultrasonographic assessment of the equine palmar tendons

**DOI:** 10.14202/vetworld.2015.208-212

**Published:** 2015-02-21

**Authors:** N. R. Padaliya, J. J. Ranpariya, Dharmendra Kumar, C. B. Javia, D. R. Barvalia

**Affiliations:** Department of Veterinary Surgery and Radiology, College of Veterinary Sciences and Animal Husbandry, Anand Agricultural University, Anand-388 001, Gujarat, India

**Keywords:** cross-sectional area, deep digital flexor tendon, horses, superficial digital flexor tendon, tendon, ultrasonography

## Abstract

**Aim:**

The present study was conducted to evaluate the equine palmar tendon by ultrasonography (USG) in standing the position.

**Materials and Methods:**

USG of palmar tendons was performed in 40 adult horses using linear transducer having frequency of 10-18 MHz (e-soate, My Lab FIVE) and L52 linear array transducer (Titan, SonoSite) with frequencies ranging from 8 to 10 MHz. Palmar tendon was divided into 7 levels from distal to accessory carpal bone up to ergot in transverse scanning and 3 levels in longitudinal scanning.

**Results:**

The USG evaluation was very useful for diagnosis of affections of the conditions such as chronic bowed tendon, suspensory ligament desmitis, carpal sheath tenosynovitis and digital sheath effusions. The mean cross-sectional area (cm^2^) of affected tendons was significantly increased in affected than normal tendons. The echogenicity was also found reduced in affected tendons and ligaments along with disorganization of fiber alignment depending on the severity of lesion and injury.

**Conclusion:**

USG proved ideal diagnostic tool for diagnosis and post-treatment healing assessment of tendon injuries in horses.

## Introduction

Horse is a large land mammal notable for its speed, strength and endurance. The horse is extremely well adapted to traveling long distances with great efficiency as described by Budiansky [1], Ultrasonography (USG) has enabled the practitioner to diagnose the presence of tendon and ligament injury, characterize the type of injury and quantitate its severity. Tendon injuries including the superficial digital flexor tendon (SDFT) are common seen in horses and healing time varies depending on aspects such as the age, general health, the tendon involved and the severity of the injury described by Thorpe *et al*. [2]. USG is currently being used to assess tendon healing and is becoming an essential part of the rehabilitation program for a horse recovering from a tendon or ligament injury as opined by Maoudifard [3], tendon injury is an important cause of wastage in racing due to a high frequency of re-injury and a less well defined adverse effect on performance. There is considerable interest in the monitoring of tendon health in racehorses so that training can be altered to prevent major injury. Diagnostic USG can identify tendon pathology and is manifested by changes in cross-sectional area (CSA), echogenicity, fiber pattern, shape, margination and position as per Davis *et al*.[4], US is currently an integral tool for the evaluation of equine tendons and ligaments though it is not without its limitations Rantanen [5].

The aim of the present study was to compare the transverse and longitudinal USG of normal and affected palmar tendons.

## Materials and Methods

### Ethical approval

All clinical cases in this research were examined and diagnosed as per standard examination and diagnostic procedure without harming or discomforts to animals.

### Animal selection

For clinical study animals brought to the Department of Surgery or College Clinic for treatment and a Government Horse Breeding Farm, Junagadh as well as Mounted Police Force, Anand were selected.

### Patient preparation

**Figure-1:**
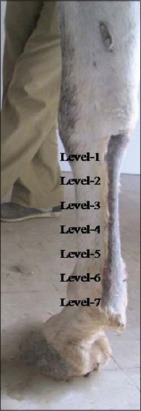
Limb position and different levels of scanning at metacarpal region.

With the owners’ consent, both forelimbs of the horse between the carpus and metacarpophalangeal joints were prepared for USG examination by shaving. The area was sprayed with spirit or 70% alcohol to degrease the skin. Echolucent gel (scan care gel) was applied to the whole area of the scanning and massaged thoroughly. The area of interest of both forelimbs between distal border of accessory carpal bone (DACB) and the ergot was divided into seven zones (Figure-1). Distal base of the accessory carpal bone was fixed as ‘0’ followed by division of the length of the tendon at about 4 cm intervals, into 7 zones and marked using paper adhesive tape to maintain the uniformity and systematic approach in the procedure of tendon scanning, examination and evaluation of the results. For marking the zones, a centimeter measuring tape was used. All animals were approached from the left side and both forelimbs were scanned from the left side, while recording the sonograms the limb was kept under complete weight bearing condition otherwise ligaments and tendons change in shape, size and echogenicity if not loaded by full weight bearing.

### Ultrasound equipment and probe

Ultrasound scans were performed with portable machine (Titan, SonoSite) equipped with Probe L52, linear array transducer with frequency range from 8 to 10 MHz and (e-soate, My Lab FIVE) machine equipped with linear transducer having frequency of 10-18 MHz. The images were acquired at the depth 3.5-6 cm for visualization of all tendons and ligament of palmar and planter regions. The equipment was set on the musculoskeletal scanning frequency pre-programmed according to the probe attached. Animals included in the study were restrained with the help of trainer or owner in standing the position. Probe was placed exactly perpendicular to the longitudinal axis of the tendon particularly for transverse imaging and excessive pressure was avoided which altered the shape and size of the underlying soft tissue structures thereby error in the CSA measurements were minimized.

USG anatomy of the palmar aspect of the metacarpal region was assessed at seven levels (1A, 1B, 2A, 2B, 3A, 3B, 3C) through transverse scanning and at 3 levels on longitudinal scanning.

CSA of SDFT and deep digital flexor tendon (DDFT) was measured using the caliper function available in the equipment. The sonograms acquired were also subjected to a thorough evaluation regarding shape, size, echogenicity and fiber alignment of tendons and ligaments.

### Statistical analysis

Data generated were statically analyzed for calculation of mean, standard error, standard deviation and paired t-test of CSA of the SDFT and DDFT.

## Results

**Figure-2:**
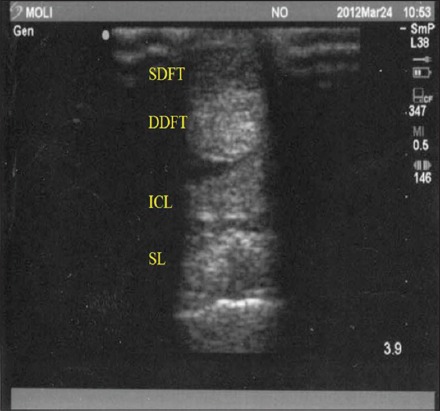
Normal transverse ultrasonogram of the equine palmar tendon. SDFT = Superficial digital flexor tendon, DDFT = Deep digital flexor tendon, ICL = Inferior check ligament, SL = suspensory ligament.

**Figure-3:**
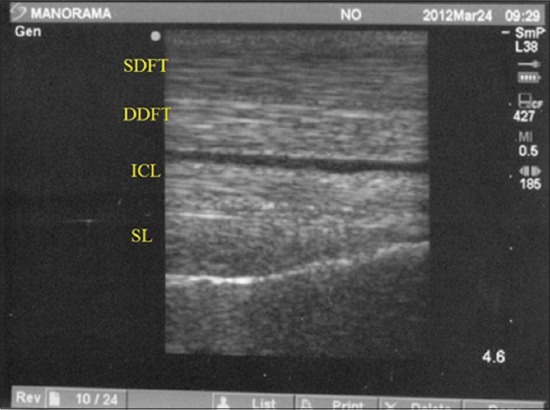
Normal longitudinal ultrasonogram of the equine palmar tendon. SDFT = Superficial digital flexor tendon, DDFT = Deep digital flexor tendon, ICL = Inferior check ligament, SL = suspensory ligament.

**Figure-4:**
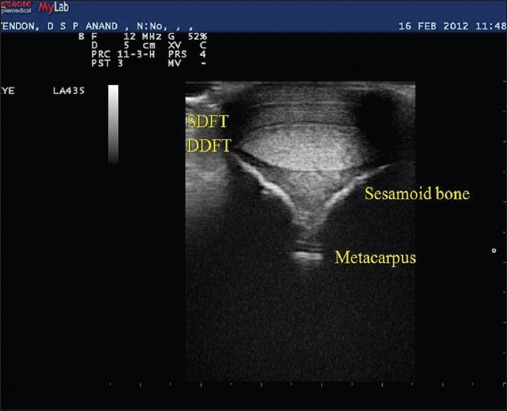
Normal transverse ultrasonogram of the equine palmar tendon. SDFT = Superficial digital flexor tendon, DDFT = Deep digital flexor tendon.

The tendons and ligaments of the metacarpal region of all the equines were evaluated ultrasonographically. Out of forty, twelve horses were found having problem of tendon and ligament unilaterally or bilaterally. The CSA of both SDFT and DDFT were calibrated. All structures of the metacarpal and metatarsal like tendons (SDFT and DDFT) and ligaments (Inferior check ligament [ICL] and Suspensory ligament [SL]) were observed for their echogenicity and fiber alignment of the left and right limbs in all horses. SDFT was the first structure to be visualized in transverse as well as longitudinal scans. The SDFT was visualized sonographically in its entire length in the metacarpal region. The DDFT was the second structure noticed in the transverse and longitudinal scans, also visualized entirely throughout the metacarpal length in the forelimbs of all animals. Accessory ligament (AL) or ICL was the third structure that appeared in both scanning planes in the proximal metacarpal region up to level 16 cm distal to the DACB. SL was the fourth and last structure just above the metacarpal bone formed while scanning in both planes. The origin of the SL was clearly noticed just distal to the origin of AL at the DACB (Figures-2 and -3). Lateral and medial branches of SL was clearly noticed in level 3A and 3B (level 5 and 6) in transverse scanning and in last level palmar ligament and two echogenic convex line of sesamoidean bones visualized (Figure-4).

### CSA of normal equine palmar tendons

The mean CSA (cm^2^) value between breeds as well as within breeds was significantly higher in DDFT than SDFT. The mean CSA (cm^2^) for left limb DDFT in Kathiawadi horses was 0.88±0.07 cm^2^and mean CSA for left limb SDFT in Kathiawadi was 0.58±0.04cm^2^. Likewise, in Marwadi horses the mean CSA of the left limb DDFT (0.86±0.01cm^2^) was higher than mean CSA of the left limb SDFT (0.55±0.04cm^2^). Further, there was a significant difference in CSA (cm^2^) values between Kathiawadi and Marwadi horses for both SDFT and DDFT (Table-1).

**Table-1:** CSA (cm^2^) of normal equine digital flexor tendons in various breeds mean±SE.

Breed	Mean±SE CSA (cm^2^)	

	SDFT area	DDFT area
Kathiawadi left limb normal	0.58±0.04*	0.88±0.07*
Marwadi left limb normal	0.55±0.04	0.84±0.07
Kathiawadi right limb normal	0.59±0.04*	0.89±0.07*
Marwadi right limb normal	0.56±0.04	0.86±0.07
Kathiawadi left limb normal	0.58±0.04	0.88±0.07
Kathiawadi right limb normal	0.59±0.04	0.89±0.07
Marwadi left limb normal	0.55±0.04	0.84±0.07
Marwadi right limb normal	0.56±0.04	0.86±0.07

* Means in the raw bearing different superscripts differs significantly (P≤0.05), SDFT=Superficial digital flexor tendon, DDFT=Deep digital flexor tendon, CSA=Crosssectional area, SE=Standard error

### CSA of abnormal equine palmar tendons

The CSA (cm^2^) value for left limb SDFT in Kathiawadi was 0.74±0.05 cm^2^ and Marwadi horses was 0.74±0.05 cm^2^. Similarly CSA value of right limb SDFT of Kathiawadi was 0.77±0.04 cm^2^ and Marwadi horses was 0.72±0.05 cm^2^.

Mean CSA of abnormal DDFT of left and right limb of Kathiawadi horses was 1.05±0.08 and 1.07±0.09 cm^2^ whereas, in Marwadi horses was 0.99±0.06 and 1.00±0.06 cm^2^, respectively.

There was highly significant difference between CSA of normal (0.58±0.04 cm^2^) and abnormal (0.74±0.05 cm^2^) left limb SDFT as well as between normal (0.59±0.04 cm^2^) and abnormal (0.77±0.04 cm^2^) right limb SDFT.

### Echogenicity

There was no echogenic difference between the breeds and particular tendon and ligament of normal horses. In transverse and longitudinal scanning SDFT and DDFT had homogenous and echogenic appearance but DDFT was more echogenic than SDFT (Figure-2). Echogenicity remained consistent at all levels for right and left forelimbs. SL normally was having coarser echotexture (heterogenous) and small hypoechoeic regions. SL was clearly seen in longitudinal scanning up to level L2 and afterward it divides into lateral and medial branches those could not be seen in longitudinal scanning. At longitudinal level 3 (L3) palmar pouch of the metacarpophalangeal joint was seen as well as palmar or annular ligament was seen in level 7 in transverse scanning and echogenicity was lesser than that of SDFT and DDFT. Two echogenic convex lines were found in the last level of transverse scanning.

The USG obtained in this study revealed AL as most echogenic structure of all four structures visualized in the metacarpal region of horses. Further, SDFT was less echogenic than DDFT, whereas SL was slightly hyperechoic with coarser echotexture (Figures-2 and -3).

### Fiber alignment

The tendon or ligament fiber alignment differed in transverse and longitudinal scans. In the longitudinal scans the tendons had uniformly linear fiber alignment running parallel to the long axis along the metacarpal bone, whereas in transverse scans the cross-sectional tendons revealed stippled fiber appearance. However, in SL particularly a slightly coarser linear fiber pattern was present. The fiber alignment of tendons and ligaments of the metacarpal regions were scrutinized and graded according to the severity of the lesion into four grades ranging from 0 to 3.

## Discussion

USG of equine palmar tendons was performed in standing position using linear array transducer with a frequency range from 8 to 10 MHz. Area was shaved, and gel was applied at metacarpal region. Satisfactory tendon sonograms were achieved as per this method. The preparation of patient was carried out according method described by Hodgson and Rose [6], Sande *et al*. [7], Reef [8], Whitcomb [9], Pickersgill [10] and Karlin [11]. The area of interest was scanned, and images were captured in dual mode at 7 levels from distal base of accessory carpal bone up to ergot which gave complete details of the area under examination.

The SDFT was the first structure to be visualized in transverse as well as longitudinal scans followed by DDFT, AL and SL in the present study. The first two (SDFT and DDFT) structures were imaged entirely throughout the metacarpal length while AL fused with DDFT just below the mid-metacarpal region and under the body of the SL bifurcated into its lateral and medial branches. The branches of SL were noticed clearly at about 24 cm DACB. These observations were found in agreement with the findings of Reef [8] and Craychee [12].

Study proved that measurement of CSA is a satisfactory method to evaluate the status of flexor tendons and ligaments of the metacarpal or metatarsal region in equines. Although echogenicity, size, shape and fiber alignment of the tendon and ligaments were the basis for the assessment of tendon or ligament injury; whereas, measurement of CSA was the most valid and accurate technique for detection of the tendon or ligament injury as opined by Maoudifard [3], Reef [8], Craychee [12], Nerurkar [13] and Reis and Baccarian [14].

In the present study, the mean value of CSA for left limb SDFT was found to be 0.58±0.01 cm^2^ and right limb was 0.59±0.04 cm^2^ for Kathiawadi horses used mainly for breeding purpose at HBF Junagadh. CSA ≤0.8-1.0 cm^2^ in the forelimb of the SDFT in normal thoroughbred and Standard bred and 0.6-0.8 cm^2^ in more fine boned breeds such as Arabian and normal tendon CSA may be slightly be larger in draft or large breeds such as Warmblood breeds, depending upon the size of the horse and the portion of the tendon under investigation as reported by Maoudifard [3] and Reef [15].

No statistically significant difference in CSA for any of the forelimb flexor tendons measured in three groups *viz*., heavy horses, Thoroughbred horses and ponies as reported by Smith *et al*. [16]. The slightly hour-glass shape of SDFT and DDFT was defined by USG CSA measurements, with smallest CSA observed in the mid-metacarpal region (levels 2 and 3). Measurement of CSA is a satisfactory method to evaluate the status of flexor tendons and ligaments of the metacarpal or metatarsal region in equines. Although echogenicity, size, shape and fiber alignment of the tendon and ligaments were the basis for the assessment of tendon or ligament injury, measurement of CSA was the most valid and accurate technique for detection of the tendon or ligament injury as evaluated by Maoudifard [3], Nerurkar [13] and Reis, Baccarian [14] and Dehghan *et al*. [17].

Mild enlargement of the tendon and ligament with a diffuse decreased echogenicity of tendon or ligament and preservation of fiber alignment is frequently detected in horses with newly discovered tendonitis or desmitis. This is an early indication of tendinitis, ligament inflammation or injury and often preceded the development of a bowed tendon or torn ligament. Fiber damage to the tendon or ligament appears sonographically as an anechoic or hypoechoic regions lacking parallel fiber pattern as described by Reef [15], and further Agut *et al*. [18], opined tendon and ligament of the metacarpal region in pure standard bred horses showed similar relative echogenicity but smaller CSA when compared to other breeds. However, Reimer [19] described enlarged hypoechoic lesion in cross sectional plane, and transverse plane in AL of SDFT and fiber alignment was poor in affected tendon region in the majority of cases. Similarly, in the present study more than 50% fiber alignment damage was found in affected cases. Usg can also helpful for healing assessment in tendon and ligament disorders like bowed tendon, desmitis and tendinitis and the tendon sheath effusion after various treatments Riccardi [20] and Ramirez [21].

## Conclusions

Mean CSA of SDFT and DDFT for Kathiawadi and Marwadi horses of both limbs was significantly different. Highly significant difference was observed between normal and affected SDFT and DDFT tendons in both breeds. Alterations in CSA in the affected tendons were related to the extent of injury, and thus CSA proved as a reliable parameter for the diagnosis of tendon affections in equines. Echogenicity and fiber alignment were altered in the affected tendons and ligaments remarkably.

## Authors’ Contributions

NRP, DK and JJR have performed all clinical cases in college and field under guidance of DRB. NRP, DK and CBJ have conducted the research, analyzed and kept a due record of the data. Statistical analysis of data conducted by JJR and DK. Manuscript was framed and drafted by NRP & JJR under the aegis of DRB. All authors read and approved the final manuscript.
